# Can Surgery Be Avoided? Exclusive Antibiotic Treatment for Pelvic Actinomycosis

**DOI:** 10.1155/2017/2907135

**Published:** 2017-02-16

**Authors:** M. P. Ruiz, E. M. Williams, C. M. Markey, A. M. Johnson, P. B. Morales-Ramirez

**Affiliations:** ^1^Department of Obstetrics and Gynecology, University of Missouri Kansas City, Kansas City, MO, USA; ^2^University of Missouri Kansas City School of Medicine, Kansas City, MO, USA

## Abstract

Pelvic actinomycosis is an uncommon, slowly progressing granulomatous infection that has been associated with the presence of intrauterine devices. Due to its unspecific clinical and radiologic findings, it can mimic pelvic or intra-abdominal malignancy leading to mutilating surgery of high morbidity. Rarely, diagnosis is made preoperatively and in most cases surgical intervention is necessary. The patient in our case is a 42-year-old female with an IUD for 15 years diagnosed with pelvic actinomycosis. Patient was uniquely diagnosed preoperatively through paracentesis and treated conservatively with prolonged antibiotic therapy and without any type of surgical intervention. Follow-up at 1 year showed almost complete radiologic resolution of the inflammatory mass, nutritional recovery, and absence of symptoms. Pelvic actinomycosis can be successfully diagnosed and treated medically without surgical interventions.

## 1. Introduction

Pelvic actinomycosis is a rare indolent disease caused most frequently by* Actinomyces israelii*, an anaerobic gram positive bacillus that is part of the normal flora of the oropharynx, gastrointestinal tract, and female genital tract [1–5]. The infection is characterized by suppurative and granulomatous inflammation, contiguous spread, and the formation of colonies described macroscopically as sulfur granules [2, 3, 6]. It can become pathogenic when mucosal integrity is compromised or in the presence of a foreign body, like an IUD [2]. Pelvic actinomycosis represents approximately 3% of human actinomycosis infections [1]. A definitive diagnosis requires a positive anaerobic culture or histologic identification of actinomyces sulfur granules. The infection typically manifests as a unilateral tuboovarian abscess but can also present as an ovarian tumor or retroperitoneal mass that mimics a pelvic or intra-abdominal malignancy that requires extensive surgical intervention [6]. For those reasons, the diagnosis is made in most of the cases intraoperatively. The case presented demonstrates that preoperative diagnosis of pelvic actinomycosis can be achieved through CT-guided paracentesis if there is high clinical suspicion and that conservative management with prolonged antibiotics can result in clinical and radiologic resolution.

## 2. Case Presentation

A 42-year-old Caucasian female, gravida 3 para 1 (1 spontaneous vaginal delivery and 2 elective abortions), was admitted with a 2-month history of diffuse abdominal pain, decreased appetite, nausea, vomiting, abdominal distention, weakness, and a twenty-pound unintentional weight loss. Her history was significant for a twenty-eight-pack-year tobacco use and a copper IUD placed approximately fifteen years earlier.

On admission, the patient was found to be cachectic, tachycardic, and tachypneic but afebrile. Her abdomen was moderately distended, tender to palpation throughout but without acute peritoneal signs. It was diffusely tympanic but dull over bilateral lower quadrants, with hypoactive bowel sounds. External genitalia and vagina were normal. Cervix was parous in appearance with two strings visualized from the cervical os; IUD was removed and sent for microbiologic studies. Uterus was difficult to palpate secondary to pain and adnexal tenderness. Adnexa were diffusely tender with bilateral fullness. Rectal exam showed normal tone and normal rectovaginal septum with fullness palpated anteriorly.

The patient's initial laboratory evaluation showed marked leukocytosis (WBC: 29,000), anemia (Hgb: 10.9), hypoalbuminemia (albumin: 2.2), and mildly elevated CA 125 (54). AST/ALT, CEA, and CA 19-9 were normal. No infectious disease was found per evaluation via urine and blood cultures, gonorrhea, chlamydia, vaginitis panel (testing for trichomoniasis, candida, and bacterial vaginosis), tuberculin test, HIV, or hepatitis B and hepatitis C.

**Figure 1:**
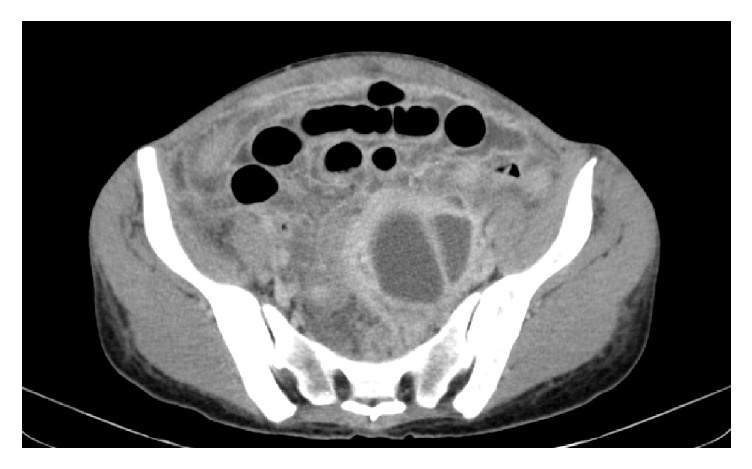
CT of pelvis demonstrating multiloculated abscess and dilated bowel loops.

CT of the abdomen demonstrated marked bowel distention and a complex cystic mass within the left pelvis with enhancing septations measuring 8.2 × 6.7 × 9.8 cm, retroperitoneal lymphadenopathy, and small ascites with peritoneal enhancement. Large distended bowel loops surrounded the heterogeneous lesion peripherally (Figure 1).

**Figure 2:**
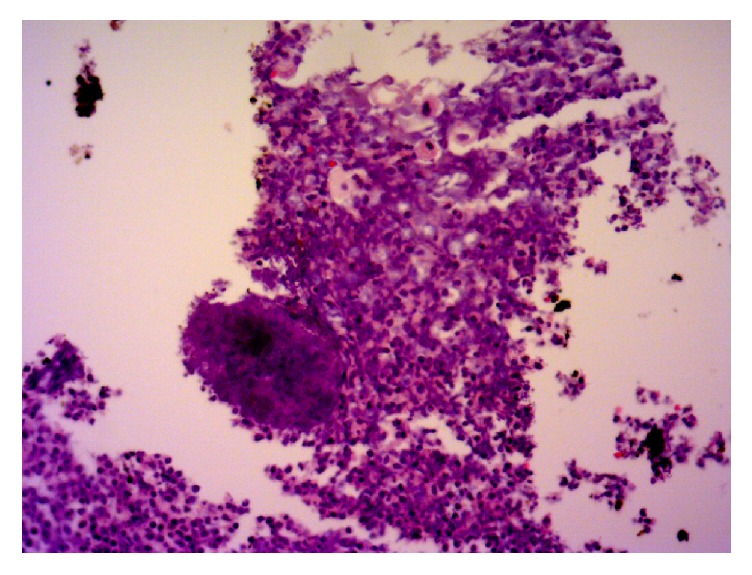
Sulfur granules from peritoneal aspiration, 40x.

**Figure 3:**
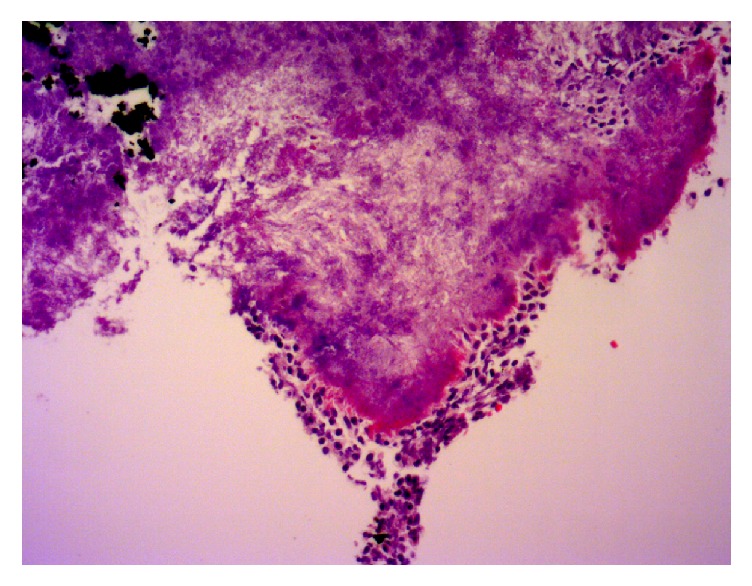
Sulfur granules from IUD, 40x.

Parenteral antibiotics (Meropenem and Vancomycin) were started for coverage of pelvic abscess of unknown etiology with noted improvement in leukocytosis after 72 hours. Nasogastric tube was placed for upper GI decompression and control of emesis for suspected partial small bowel obstruction. Total parenteral nutrition was also started to improve her malnourished state. Gynecologic oncology was consulted and concluded that the likelihood of carcinomatosis was very low based on the patient's age, absence of omental caking or peritoneal implants on CT, and only mildly elevated CA-125. In the presence of a neglected IUD for 15 years with low suspicion for malignancy, actinomycosis was a likely diagnosis. CT-guided diagnostic paracentesis was performed after bowel decompression was achieved with NG tube. Purulent peritoneal fluid was obtained which was negative for malignancy, yet positive for* Actinomyces* (Figure 2). Copper IUD was removed and culture grew* Micromonas micros* and* Actinomyces* (Figure 3).

One week after initiation of antibiotic therapy, repeat CT of abdomen and pelvis showed stable to slightly improved fluid collections compared to prior exams. As final laboratory diagnosis was achieved, Vancomycin and Meropenem were discontinued after 8 and 11 days of treatment, respectively.

The patient was started on Ampicillin-Sulbactam 3 g IV every 6 hours, which helped control her leukocytosis. On day five of the Ampicillin-Sulbactam, AST and ALT values were 209 and 148, respectively, and patient was diagnosed with transaminitis secondary to the Ampicillin-Sulbactam. The Ampicillin-Sulbactam was discontinued, which improved the transaminitis, and the patient was started on Penicillin 3 million units IV every 4 hours, which was continued for a total of 4 weeks.

After 3 weeks of in-hospital management, patient was tolerating regular diet with regular bowel movements and improved pain control. Patient was discharged home with close outpatient follow-up arranged. At her outpatient follow-up four weeks later, she was transitioned to Penicillin 500 mg PO four times daily. Patient continued Penicillin orally for 1 year, during which she was gaining the strength and weight back with assistance of nutrition and occupational therapy professionals.

**Figure 4:**
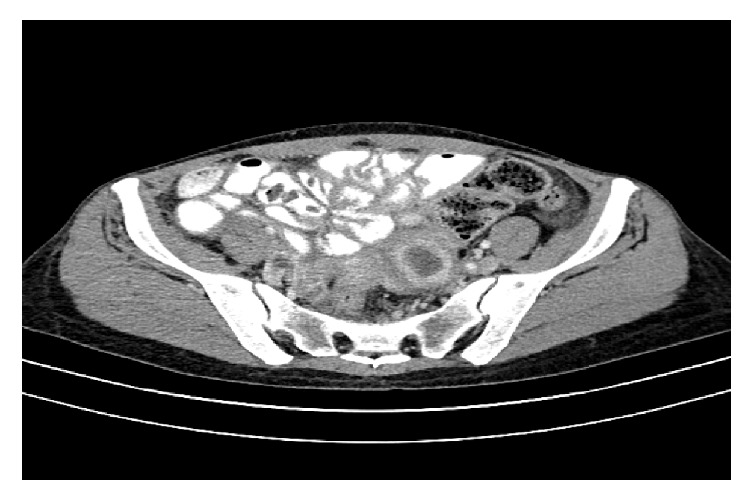
CT of pelvis demonstrating reduction of adnexal mass and decompressed bowel loops at 1 year of treatment.

Repeat CT of abdomen and pelvis 1 year after initiation of treatment demonstrated complete resolution of bowel distention, ascites, and adenopathy; the left adnexal mass had more than 50% size reduction, measuring 3.9 × 3.2 cm (Figure 4). The patient was found to have complete resolution of pain and abdominal distention, as well as 21 lbs weight gain since treatment was initiated.

## 3. Discussion

Pelvic actinomycosis has a characteristic invasion pattern that causes an intense inflammatory reaction with subsequent risk of intestinal obstructions, strictures, and even mass effect leading to ureteral obstruction. Acute surgical intervention is usually performed due to very unspecific clinical presentation with concern for malignancy or acute abdomen. This approach frequently leads to extensive removal of reproductive or gastrointestinal organs with greatly increased morbidity. If actinomycosis is highly suspected or diagnosed preoperatively, delayed surgical intervention after prolonged antibiotic therapy and metabolic support is the preferred approach.

Diagnosing pelvic actinomycosis before surgical intervention is extremely uncommon. Although this chronic infection can manifest as tuboovarian abscesses, in many cases it mimics pelvic or intra-abdominal malignancy, cervix carcinoma, or colorectal cancer, leading to surgical intervention in almost all cases [1]. To our knowledge, only four reported cases of pelvic actinomycosis diagnosed preoperatively exist [5–10].

In our case, diagnosis was successfully achieved through CT-guided paracentesis instead of the commonly performed surgical approach. The patient was then treated exclusively with noninvasive medical management. We found only six other reports of preoperative diagnosis of pelvic actinomycosis that support exclusive medical management in the absence of a surgical abdomen [5–10]. As long as no acute abdominal signs are present, diagnostic paracentesis should be considered. Although distended bowel loops make it difficult for imaging guided biopsy, nasogastric decompression can assist. Paracentesis becomes a very important diagnostic tool, as in our case, pointing out the causative agent via histologic and microbiologic studies. Additionally, the absence of malignant cells in the peritoneal fluid makes carcinomatosis unlikely. Further evaluation by removal of IUD and pap smear collection can also support the diagnosis. Pap smear is a simple diagnostic aid of greater sensitivity than vaginal cultures, with accuracy of up to 69% [6]. Once diagnosis is confirmed, exclusive medical management with long term use of antibiotics and close follow-up is an acceptable approach which could prevent the need for extensive and risky surgical interventions in patients with pelvic actinomycosis.
